# Utility of the Inflammatory Bowel Disease Serology, Genetics, and Inflammation (IBD-SGI) Panel in the Evaluation of Atypical Ulcerative Colitis With Active Nonsteroidal Anti-Inflammatory Drug Use: A Case Report

**DOI:** 10.7759/cureus.114025

**Published:** 2026-08-05

**Authors:** Landys Z Guo, Ren Bryant, Krista Perry

**Affiliations:** 1 Internal Medicine, Charleston Area Medical Center (CAMC) Health Education and Research Institute, Charleston, USA; 2 Internal Medicine, University of Kentucky HealthCare, Lexington, USA; 3 Primary Care, Internal Medicine, and Geriatrics, University of Kentucky HealthCare, Lexington, USA

**Keywords:** hematochezia, ibd-sgi panel, inflammatory bowel disease, nsaid, nsaid-induced colitis

## Abstract

Hematochezia has a broad differential, including nonsteroidal anti-inflammatory drug (NSAID)-induced, infectious, and inflammatory colitis. Colonoscopy with biopsy is primarily used to evaluate suspected colitis or inflammatory bowel disease (IBD) in patients with hematochezia, rather than to diagnose all causes of hematochezia. While the inflammatory bowel disease serology, genetics, and inflammation (IBD-SGI) blood panel test has been studied and marketed by laboratories, it is not widely implemented in routine clinical practice due to the restriction of current clinical guidelines, lack of FDA clearance/approval, and limited medical insurance coverage. Thus, we report a case of the utility of the IBD-SGI panel in the evaluation of atypical ulcerative colitis (UC) with active NSAID use. A 39-year-old man with hematochezia, diarrhea, and chronic high-dose NSAID use developed anemia, elevated fecal calprotectin, and endoscopic evidence of inflammation. Biopsies by colonoscopy showed focal colitis with features suggestive of both NSAID injury and IBD. To provide additional diagnostic support in this challenging case, an IBD-SGI blood panel was used. When interpreted with the patient’s clinical presentation, elevated fecal calprotectin level, colonoscopic findings, and histopathologic features, the panel results supported a diagnosis of UC. Based on the overall clinic-pathological assessment, including continuous circumferential inflammation on colonoscopy and histopathologic evidence of chronic IBD, the patient was diagnosed with UC and treatment with vedolizumab was initiated. Anemia attributed to hematochezia was also managed. At the three-month follow-up, the patient had resumed his daily activities and returned to work. In the setting of overlapping features of UC and possible NSAID-associated colitis, the IBD-SGI panel provided adjunctive diagnostic information. This case highlights the panel’s potential value as a supportive tool when endoscopic biopsy findings are inconclusive and multiple possible etiologies coexist.

## Introduction

Inflammatory bowel disease (IBD), such as ulcerative colitis (UC), is suspected in patients with hematochezia, increased stool frequency, and fecal urgency. Diagnostic evaluation includes stool testing for infectious colitis, colonoscopy, and abdominal CT or MRI when endoscopic findings are inconclusive [1]. Typical endoscopic findings of active UC include continuous, circumferential inflammation beginning in the rectum, with erythema, loss of vascular pattern, granularity, friability, erosions, and superficial ulceration. Histological findings may include crypt architectural distortion, basal plasmacytosis, chronic lymphoplasmacytic inflammation, cryptitis, and crypt abscesses. In severe or longstanding disease, deep ulcerations, inflammatory pseudopolyps, mucosal atrophy, and loss of haustration may occur. Epithelial dysplasia may develop as a chronic complication. Colonic strictures are uncommon and should prompt evaluation for colorectal neoplasia, chronic complications, or an alternative diagnosis such as Crohn’s disease.

Although nonsteroidal anti-inflammatory drugs (NSAIDs) have systemic anti-inflammatory effects and have been associated with potential chemopreventive effects against colorectal neoplasia, they can also directly injure the colonic mucosa and produce inflammatory changes that mimic IBD [1]. NSAID-associated colitis may present with erosions, ulceration, and mucosal inflammation; therefore, the patient’s NSAID exposure was considered clinically relevant in the differential diagnosis.

The inflammatory bowel disease serology, genetics, and inflammation (IBD-SGI) panel is a multimarker blood test that combines serologic, genetic, and inflammatory biomarkers to provide adjunctive information when differentiating IBD from non-IBD conditions and UC from Crohn’s disease [2]. Manufacturer-reported sensitivities for IBD, UC, and Crohn’s disease are 74%, 98%, and 89%, respectively, with corresponding specificities of 90%, 84%, and 81% [3]. However, these performance estimates have not been clearly supported by an independently cited validation study, and performance measures for directly distinguishing UC from Crohn’s disease were not reported [3]. Therefore, the panel should be interpreted as adjunctive evidence rather than as a stand-alone diagnostic test. Here, we present a case illustrating the possible adjunctive role of the IBD-SGI panel when standard diagnostic findings are inconclusive and NSAID-associated colitis and IBD are both considered.

This case report was previously presented as a poster at the 2025 American College of Gastroenterology (ACG) Annual Scientific Meeting on October 26, 2025.

## Case presentation

A 39-year-old Caucasian male reported hematochezia, increased stool frequency, bloating, abdominal cramping, and loose stools. He denied postprandial abdominal pain, night sweats, weight loss, rash, oral or skin ulcers, and melena. At a visit about two years before the current presentation, the patient reported taking Aleve (naproxen) 250 mg once or twice monthly for generalized joint pain over several years. The available record did not document daily NSAID use, concurrent aspirin or other over-the-counter analgesic use, or whether naproxen use continued until the current presentation. Given the intermittent and incompletely characterized exposure, NSAID-associated colitis was considered a possible but unconfirmed competing diagnosis.

The patient had no family history of colon cancer, but the patient’s father had colon polyps, and the patient’s paternal grandmother had gastric/pancreatic cancer. The patient consumed two beers and two shots a week, with no tobacco use. At the initial evaluation, the C-reactive protein (CRP) level was 3.7 mg/L (reference range: <8 mg/L) and the erythrocyte sedimentation rate was 8 mm/hour (reference range: <15 mm/hour), both within their respective reference ranges. The mean corpuscular volume was 85 fL, within the reference range of 80-100 fL. The hemoglobin level was lower at 13.6 g/dL (reference range: 13.7-17.5 g/dL), accompanied by a low ferritin level of 18 ng/mL (reference range: 20-400 ng/mL), supporting mild iron deficiency in the setting of hematochezia. Fecal calprotectin was markedly elevated to 1,080 µg/g (reference range: <49 µg/g), providing objective evidence of intestinal inflammation.

**Figure 1:**
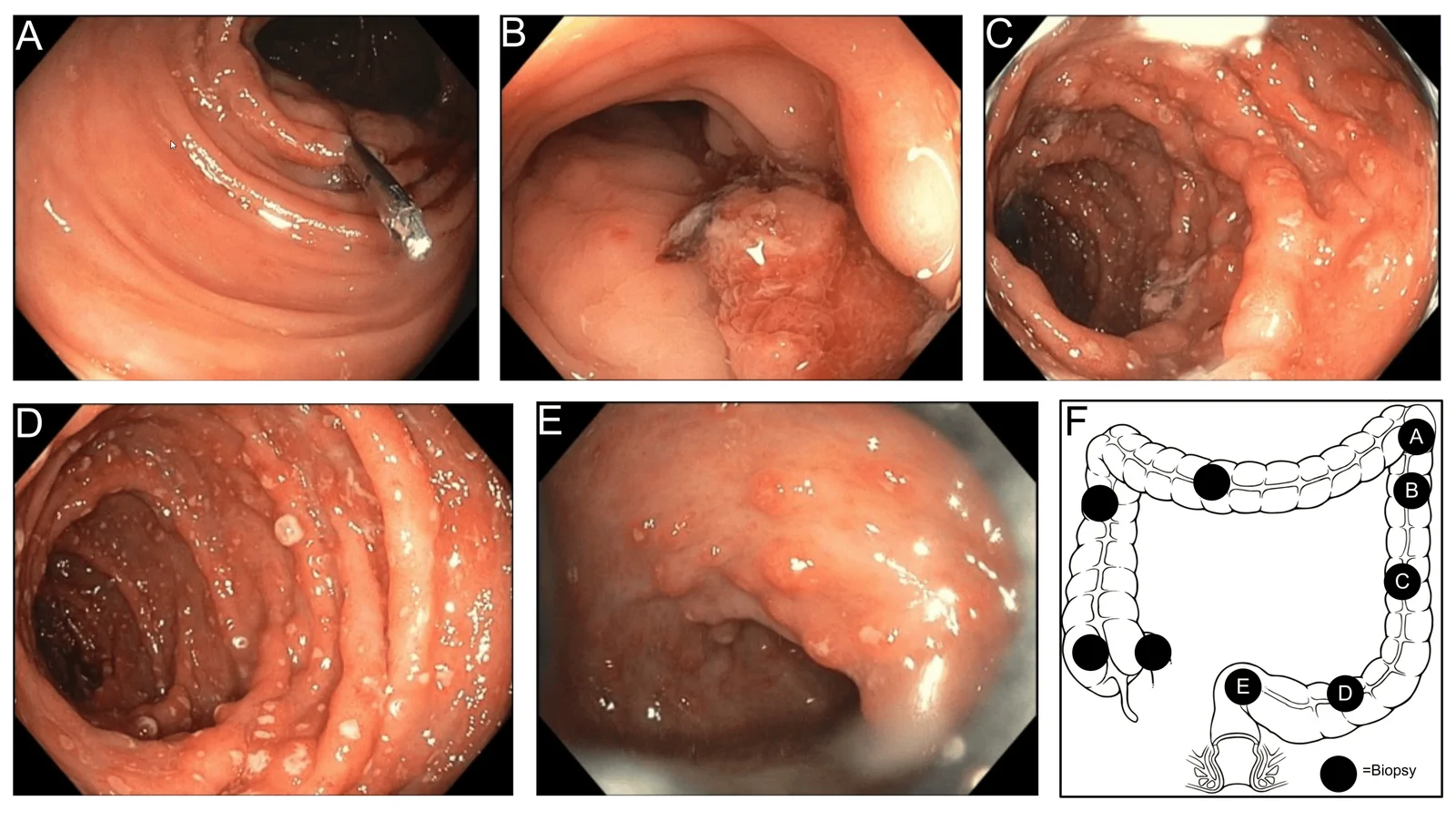
Colonoscopy findings and biopsy locations. (A) Splenic flexure showing the proximal extent of inflammation, with mild mucosal erythema and loss of the normal vascular pattern. (B) Descending colon showing erythematous, edematous, and friable mucosa with surface exudate. (C) Descending colon showing diffuse circumferential inflammation with mucosal erythema, edema, granularity, and superficial ulceration. (D) Sigmoid colon showing continuous circumferential inflammation with marked erythema, granularity, loss of vascular pattern, and scattered surface exudate. (E) Rectosigmoid junction showing pronounced mucosal erythema, edema, friability, and irregularity. (F) Schematic representation of the colon indicating the locations from which endoscopic images and biopsy specimens were obtained. The schematic was adapted from the University of Kentucky colonoscopy medical report provided to the patient.

Initial colonoscopy revealed three pedunculated polyps and continuous circumferential inflammation extending proximally to the splenic flexure (Figure 1). Colonoscopy-guided biopsies from the inflamed segment demonstrated focal chronic active colitis with features of chronicity and activity, including crypt architectural distortion, infrequent crypt abscesses, Paneth cell metaplasia, and eosinophilic cryptitis. The pathologist considered these findings nonspecific, with overlapping features of IBD and medication-associated injury, including possible NSAID-related injury. Because neither etiology was definitively favored on histopathology alone, the biopsy findings did not independently establish UC, and clinic-pathological correlation was recommended.

Stool cultures, *Clostridioides difficile* testing, ova and parasite testing, and a gastrointestinal pathogen panel were not documented. Infectious colitis was considered less likely given the absence of documented systemic infectious features, the continuous circumferential pattern of inflammation on colonoscopy, and histopathologic evidence of chronicity, including crypt architectural distortion and Paneth cell metaplasia. However, because a microbiologic stool evaluation was not documented, infectious colitis could not be definitively excluded, representing a limitation of this case.

To provide additional supportive evidence in this diagnostically challenging case, an IBD-SGI blood panel was used. The IBD-SGI serologic results demonstrated a detected perinuclear anti-neutrophil cytoplasmic antibody (pANCA) pattern with DNase sensitivity, while both anti-*Saccharomyces cerevisiae* antibody (ASCA) measurements were below their respective reference thresholds. ASCA levels were not elevated, with ASCA IgA of <3.1 EU/mL (reference range: <9.2 EU/mL), and ASCA IgG of 9.2 EU/mL (reference range: <11.9 EU/mL). The combination of pANCA positivity, DNase sensitivity, and non-elevated ASCA levels was interpreted by the IBD-SGI panel as supportive of UC rather than Crohn’s disease.

Additionally, the IBD-SGI panel identified autophagy-related 16-like 1 (*ATG16L1*), extracellular matrix protein 1 (*ECM1*), NK2 homeobox 3 (*NKX2-3*), and variants in signal transducer and activator of transcription 3 (*STAT3*). The patient’s genotypes and corresponding variant-not-detected reference genotypes were *ATG16L1* A;G versus A;A, *ECM1* T;T versus C;C, *NKX2-3* G;G versus A;A, and *STAT3* A;G versus G;G. For *STAT3*, A;G was the patient’s genotype, whereas the G;G entry in the reference-genotype column represented the reference or comparator genotype and not an additional variant detected in the patient. These genetic findings were regarded as susceptibility markers and were not considered independently diagnostic of UC. The inflammatory marker results included elevated vascular endothelial growth factor (VEGF) at 462 pg/mL (reference range: <316 pg/mL) and serum amyloid A (SAA) at 19.7 mg/L (reference range: <7.3 mg/L) (Table 1). These elevations provided additional evidence of systemic inflammatory activity. However, because VEGF and SAA are nonspecific inflammatory markers, they were interpreted only as adjunctive findings and were not considered independently diagnostic of UC.

**Table 1:** Results from the inflammatory bowel disease serology, genetics, and inflammation (IBD-SGI) blood panel test. ELISA = enzyme-linked immunosorbent assay; ASCA IgA = anti-*Saccharomyces cerevisiae* antibody, immunoglobulin A; ASCA IgG = anti-*Saccharomyces cerevisiae* antibody, immunoglobulin G; pANCA = perinuclear anti-neutrophil cytoplasmic antibody; Anti-OmpC IgA = anti-outer membrane porin C antibody, immunoglobulin A; Anti-CBir1 IgG = anti-CBir1 flagellin antibody, immunoglobulin G; Anti-A4-Fla2 IgG = anti-A4-Fla2 flagellin antibody, immunoglobulin G; Anti-FlaX IgG = anti-FlaX flagellin antibody, immunoglobulin G; ATG16L1 = autophagy related 16 like 1; ECM1 = extracellular matrix protein 1; NKX2-3 = NK2 homeobox 3; STAT3 = signal transducer and activator of transcription 3; ICAM-1 = intercellular adhesion molecule 1; VCAM-1 = vascular cell adhesion molecule 1; VEGF = vascular endothelial growth factor; CRP = C-reactive protein; SAA = serum amyloid A

Serology results			Genetics results			Inflammatory results		
Assay (ELISA)	Result	Reference	SNP	Patient genotype	Reference genotype	Assay	Result	Reference
ASCA IgA	<3.1 EU/mL	<9.2 EU/mL	*ATG16L1*	Variant detected (A;G)	Variant not detected: (A;A)	ICAM1	0.53 µg/mL	<0.52 µg/mL
ASCA IgG	9.2 EU/mL	<11.9 EU/mL	*ECM1*	Variant detected (T;T)	Variant not detected: (C;C)	VCAM1	0.46 µg/mL	<0.66 µg/mL
Anti-OmpC IgA	<3.1 EU/mL	11.3 EU/mL	*NKX2-3*	Variant detected (G;G)	Variant not detected: (A;A)	VEGF	462 pg/mL	<316 pg/mL
Anti CBir1 IgG	23.8 EU/mL	<35.4 EU/mL	*STAT3*	Variant detected (A;G)	Variant not detected: (G;G)	CRP	6.5 mg/L	<8.0 mg/L
Anti-A4-Fla2 IgG	19.9 EU/mL	<32.4 EU/mL				SAA	19.7 mg/L	<7.3 mg/L
Anti-FlaX IgG	20.3 EU/mL	<36.0 EU/mL						
pANCA IFA perinuclear pattern	Detected	Not detected						
pANCA IFA Dnase sensitivity	DNAse sensitive	Not detected						

Treatment was initiated with vedolizumab 300 mg IV (Entyvio) for UC, along with management of anemia attributed to hematochezia. At a three-month follow-up visit for liver function monitoring, the patient reported an approximately 15-lb weight gain following induction therapy with Entyvio. Laboratory testing showed an elevated alanine aminotransferase level of 66 U/L, and liver ultrasound demonstrated mild hepatic steatosis. The patient has since resumed daily activities and returned to work.

## Discussion

The diagnosis was challenging in this case because histologic findings overlapped with both chronic IBD and possible NSAID-associated injury. Paneth cell metaplasia has a longstanding correlation with chronic inflammation and shows a strong correlation with IBD [4,5]. Yet, eosinophilic infiltration is uncommon in IBD, associated with allergic or drug-induced reactions such as NSAIDs and known to be a distinguishing factor [6]. Interestingly, when eosinophilia is present with IBD, it has associations with a milder disease course and fewer flares [7]. Because colitis associated with NSAIDs can share overlapping histologic features with UC, Crohn’s disease, and infectious colitis, a detailed clinical history is essential for identifying the underlying cause and guiding appropriate treatment. NSAIDs are known to cause crypt disarray, strictures, diaphragms with well-demarcated ulcers surrounded by normal-appearing mucosa, further illustrating conflicting pathology with the final diagnosis [6,8]. Mucosal barrier reduction from NSAIDs may cause imbalance with the intestine’s microbiome, leading to dysbiosis and inflammation. With underlying genetic risk factors, there has been some correlation with external risk factors of drug and alcohol use upsetting the threshold of gut inflammation into its pathology [9]. Some case-control studies have shown a weak correlation with chronic NSAID use, contributing to a higher risk of developing UC and microscopic colitis, with compounding risk in users over the age of 40 years [10]. However, theories of NSAIDs unmasking underlying or triggering IBD have had weak associations, with randomized controlled trials and meta-analyses pointing to NSAID use not correlating with UC or Crohn’s disease exacerbation [11-14].

Because drug-induced colitis can closely mimic IBD, adjunctive diagnostic tools may be helpful in selected cases in which the standard evaluation remains inconclusive. In such settings, the IBD-SGI panel may provide supportive evidence when interpreted together with the clinical and medication history, exclusion of infectious causes, fecal inflammatory markers, endoscopic findings, and histopathology. The IBD-SGI panel should not be used routinely or as a stand-alone diagnostic test; rather, its potential value lies in supplementing the conventional diagnostic assessment and strengthening the overall clinic-pathological interpretation in carefully selected patients.

The IBD-SGI blood panel is a commercially available, noninvasive test intended to aid diagnostic differentiation among causes of colitis by integrating serologic, genetic, and inflammatory markers [2], including to help differentiate between IBD and non-IBD, as well as to distinguish between Crohn’s disease and UC. Among the markers in the IBD-SGI blood panel, the serological test detects loss of tolerance to bacterial proteins and flagella and indicates reactivity to the endogenous microbiome patterns found in IBD pathologies. In the gene panel, *ATG16L1* prevents innate immune reactions against endogenous bacteria; ECM1 causes mucosal barrier dysfunction in UC; *NKX2-3* is a developmental gene with a role in intestinal absorption, and knockout results in bloody diarrhea; and *STAT3* plays a role in Th17/IL23 signaling [15]. The inflammatory panel utilizes cellular adhesion markers (ICAM-1, VCAM-1) for leukocyte binding and infiltration at inflammatory sites, which then make VEGF to stimulate capillary growth and perfusion. High CRP levels are correlated with increased risk of surgery and disease extent in UC [16]. Moreover, SAA has an even stronger sensitivity and specificity than CRP for predicting mucosal healing [17].

In this patient, the serologic component of the IBD-SGI panel demonstrated a detected pANCA pattern with DNase sensitivity, whereas ASCA levels were not elevated: ASCA IgA was <3.1 EU/mL (reference range: <9.2 EU/mL), and ASCA IgG was 9.2 EU/mL (reference range: <11.9 EU/mL). Within the panel’s integrated interpretation, this pANCA-positive, ASCA-non-elevated pattern was more supportive of UC than Crohn’s disease. Genetic variants were also detected in *ATG16L1*, *ECM1*, *NKX2-3*, and *STAT3*; however, these findings were interpreted as susceptibility-associated markers and were not considered specific for UC or sufficient to distinguish UC from Crohn’s disease or medication-associated colitis independently. Elevated VEGF at 462 pg/mL (reference range: <316 pg/mL) and SAA at 19.7 mg/L (reference range: <7.3 mg/L) provided additional evidence of inflammatory activity, although these markers are also nonspecific. Therefore, the IBD-SGI panel did not independently exclude NSAID-induced colitis. Rather, its UC-supportive serologic pattern and accompanying genetic and inflammatory findings were interpreted as adjunctive evidence together with the patient’s clinical presentation, medication history, continuous circumferential pattern of colonic inflammation, and histopathologic features of chronic active colitis. Overall, the IBD-SGI panel findings supported UC rather than Crohn’s disease but were not considered independently diagnostic.

Although the IBD-SGI blood panel test has been investigated and commercially marketed as a diagnostic aid for IBD, current clinical guidelines do not support routine use of genetic or serologic testing for diagnosis in standard clinical practice [1]. Lack of FDA clearance or approval [3] and limited medical insurance coverage [18] may further restrict its clinical application. Nevertheless, in carefully selected patients whose clinical, endoscopic, and histopathologic findings remain inconclusive, the IBD-SGI panel may be useful as an adjunctive diagnostic tool by integrating serologic, genetic, and inflammatory findings with the conventional evaluation.

This case illustrates the potential diagnostic value of the IBD-SGI panel in distinguishing UC from possible NSAID-associated colitis when overlapping findings create uncertainty. The diagnosis of UC was based primarily on the overall clinic-pathological assessment, including the patient’s clinical presentation, biomarker findings, continuous circumferential colonic inflammation, and histopathologic evidence of chronic active colitis. Within this context, the panel provided clinically useful supportive evidence in which pANCA positivity with DNase sensitivity and non-elevated ASCA levels favored UC over Crohn’s disease, while the detected genetic variants and elevated inflammatory markers supported underlying susceptibility and inflammatory activity. Although these findings were not independently diagnostic and did not exclude NSAID-associated colitis, they strengthened the overall interpretation in favor of UC. Therefore, the IBD-SGI panel may have a valuable selective role in supporting diagnosis when conventional findings are equivocal, while remaining complementary to clinical and medication history, exclusion of infection, endoscopy, histopathology, and guideline-directed assessment.

## Conclusions

This case highlights the diagnostic challenge of distinguishing UC from possible NSAID-associated colitis when clinical symptoms, endoscopic, and histopathologic findings overlap. The diagnosis of UC was based primarily on the overall clinic-pathological assessment, including the patient’s symptoms, biomarker findings, continuous circumferential colonic inflammation, and histopathologic evidence of chronic active colitis. In this context, the IBD-SGI panel provided adjunctive information supportive of UC rather than Crohn’s disease, but it did not independently establish the diagnosis or exclude NSAID-associated colitis. Patients presenting with symptoms suggestive of IBD should undergo careful review of NSAID exposure, with discontinuation considered when clinically appropriate, and referral for gastroenterological evaluation. In selected diagnostically inconclusive cases, the IBD-SGI panel may serve as a useful adjunct to clinical decision-making by supplementing, rather than replacing, medication history, exclusion of infectious causes, endoscopy, and histopathologic assessment.
